# Effects of Conventional and Organic Agriculture on Soil Arbuscular Mycorrhizal Fungal Community in Low-Quality Farmland

**DOI:** 10.3389/fmicb.2022.914627

**Published:** 2022-06-09

**Authors:** Jiawei Chen, Jianwei Li, Yurong Yang, Yimei Wang, Yifei Zhang, Ping Wang

**Affiliations:** ^1^State Environmental Protection Key Laboratory of Wetland Ecology and Vegetation Restoration, School of Environment, Northeast Normal University, Changchun, China; ^2^Jilin Provincial Academy of Forestry Sciences, Changchun, China

**Keywords:** arable soil, chemical fertilizer, colonization intensity, network structure, OTU diversity

## Abstract

Arbuscular mycorrhizal (AM) fungi have promising applications in low-quality farmlands all over the world, but research on their responses to conventional and organic farming systems in low-quality soil is limited. We hypothesized that the colonization activity and community diversity of AM fungi in conventional farming systems may not be lower than in organic farming on low-quality farmlands where beneficial symbiosis is required. We collected soil and maize root samples from medium to low fertility farmlands with conventional or organic farming systems in western Jilin Province, China. The colonization percentage and intensity, taxonomic and phylogenetic diversity, community composition of soil AM fungi, and soil factors were detected and compared between the two farming systems. The colonization intensity and operational taxonomic unit (OTU) taxonomic diversity on conventional farms were higher than on organic farms. *Glomus* was the most common genus on conventional farms, whereas *Paraglomus* and *Glomus* were the most common on organic farms. We also found a simpler AM fungal network structure with lower OTU phylogenetic diversity on conventional farms. Our findings suggested that though the conventional farming system resulted in different compositions and simpler structures of soil AM fungal community, there are potential diverse OTU resources currently present on conventional farms. This work has potential impacts on understanding the influence of different farming systems on soil AM fungi in low-quality farmlands and the development of efficient mycorrhizal inoculant production.

## Introduction

Soil biology is a key determinant of the effectiveness of soil nutrient resources and plays an important role in sustainable agriculture (Bender and van der Heijden, 2015). Arbuscular mycorrhizal (AM) fungi are of interest for their contribution to nutrient uptake by plants (Govindarajulu et al., 2005; Davison et al., 2015), especially in nutrient supply deficit or environmental adversities (Akiyama et al., 2005; Begum et al., 2019). Compared to other types of ecosystems, farmland usually has a higher soil nutrient content, but still requires large amounts of fertilizer inputs each year for high yields. Higher soil nutrients can weaken the mutually beneficial symbiosis formed between AM fungi and plant roots (Scullion et al., 1998; Liu et al., 2019) and decrease the colonization intensity and diversity of AM fungi (van Geel et al., 2017; Zeng et al., 2021). Therefore, several previous studies have shown that AM fungi have limited benefits for agricultural production, especially in high-yielding farmlands (Ryan and Graham, 2002, 2018). However, AM fungi are still potentially important for the large areas of low-quality agricultural land that are widely distributed worldwide. The adverse environmental conditions, such as drought or salinity, usually occur on low-quality lands (Bi et al., 2018), while mutualistic symbiosis can enhance plant growth by improving soil nutrient and water use efficiency (Begum et al., 2019). Since AM fungi have promising applications in low-quality farmland, related studies in low-quality soil conditions, such as the effects of conventional and organic farming systems on AM fungal communities, should receive more attention (Sale et al., 2015; Roy et al., 2017).

Conventional agriculture is a farming system commonly practiced throughout the world that uses large quantities of chemical fertilizers, biocides, high-yielding crop varieties, tillage, and managed irrigated systems to maximize crop yields. In practice, the application rate of chemical fertilizers has met and sometimes exceeded the needs of crop growth (Tang et al., 2008). Long-term application of chemical fertilizers can reduce spore density (Wu et al., 2011; Lin et al., 2020), species richness (Sheng et al., 2013), diversity (Oehl et al., 2004), and alter the community structure of AM fungi in agriculture soils (He et al., 2016). On the contrary, organic farming using organic amendments such as animal manure has grown rapidly in the last few decades due to the growing problems of chemical fertilizers damage to soil organisms, nutrient disorders, and environmental pollution (Yue et al., 2016). The high organic carbon content in organic amendments can promote the mycelial proliferation of AM fungi (Hart and Reader, 2002), thus increasing the inoculation capacity of AM fungi on plant roots. Therefore, some studies found that colonization activity (Qin et al., 2015; Jiang Y. J. et al., 2020) and AM fungal diversity (Bhadalung et al., 2005; Lin et al., 2012) were higher on organic farms than on conventional farms. However, we hypothesize that these effects of conventional and organic farming systems on soil AM fungi may be different in low-quality farmlands for the following reasons.

Firstly, the nutrient concentrations and release rates of chemical fertilizers applied in conventional farming systems differ from those of organic amendments applied in organic farming (Niedziński et al., 2021). When chemical fertilizers are added to the soil, AM fungi may be suppressed because the environmental nutrient content is increased by the rapid release of high nutrient concentration (Xiang et al., 2016). And the increased nutrient content in the soils in response to fertilization also reduces the mycorrhizal dependency of the crop plants. However, when soil nutrients of low-quality farmlands fall back to their original levels due to uptake by plants or loss by leaching, plant-AM fungal symbiosis may again become established widely on conventional farms. Unlike chemical fertilizers, the relatively low nutrient concentrations and slow-release rates of organic amendments applied on organic farms do not appear to inhibit the inoculation of plant roots by AM fungi. However, the negative effects of sustained nutrient release on the symbiosis should not be overlooked.

Secondly, because chemical fertilizers contain almost only one form of the nutrient, for example, the same chemical form of N, P, or K regardless of brand and manufacturer, it creates a stable environmental filter for soil organisms year after year. Therefore, it is widely believed that AM fungal diversity on conventional farms subsequently declines as some species, genera, or families are filtered out (Lin et al., 2012). However, due to polymorphism of ribosomal DNA (heterokaryosis) in individual spores of AM fungi (Pawlowska and Taylor, 2004), high genetic diversity has been found in spores and extrametrical hyphae (Bever and Wang, 2005; Tisserant et al., 2013). This suggests that even though the diversity of families, genera, or species declines, the diversity of smaller taxonomic units, such as operational taxonomic unit (OTU), may not (Rosendahl, 2008). Recent studies using high-throughput sequencing methods have also confirmed high OTU diversity in AM fungal communities in conventional farm soil (Chen et al., 2014).

Based on these two reasons, we hypothesized that the colonization activity and OTU diversity of AM fungal community in conventional farming systems will not necessarily be lower than in organic farming, especially on low-quality farmlands where beneficial symbiosis is required. To test our hypothesis, we collected soil and maize root samples from low-quality (medium-low grade farmlands in China, please see details below) maize farms in western Jilin Province, China, under conventional and organic farming management, respectively. Firstly, we assessed whether soil AM fungi activities (percentage and intensity of colonization to roots and spore density in the soil) were lower on conventional farms than on organic farms, as is commonly believed. Genus composition, taxonomy, and phylogenetic OTU diversity indices of AM fungal communities were then compared to determine whether conventional farming systems decreased AM fungal diversity on low-quality farmlands. Community composition is also a major factor influencing community function. Therefore, we created OTU networks of AM fungal communities and analyzed them with structural parameters to determine the composition of AM fungal communities on conventional and organic farms, and whether there were significant differences in their compositional characteristics. Finally, we analyzed whether the activity, diversity, and community composition of AM fungi were related to soil factors and the relative influence of each factor. This study contributes to the understanding of the effects of conventional and organic farming systems on the soil AM fungi in low-quality farmlands and promotes the rational application of AM fungal fertilizers in agro-ecosystems to restore and maintain the ecological functions of farm soils.

## Materials and Methods

### Sampling Area and Cropping History

**FIGURE 1:**
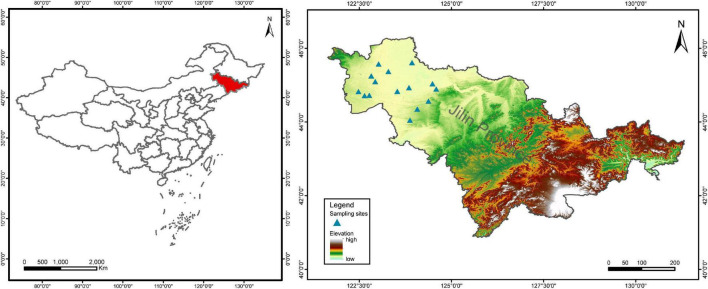
The distribution of investigated sites in western Jilin Province, China.

Jilin Province is a major maize producing area in China and belongs to the temperate maize belt of the world. This study was conducted in the semi-arid plain of western Jilin Province (Figure 1). The area has a temperate continental monsoon climate with dry springs, an average annual temperature of 4–6°C, and average annual precipitation of 350–500 mm, with precipitation concentrated in July and August (Li et al., 2005). The soil is sandy loam (Typic Hapludolls) with high salinity and low soil nutrient content (Zhu, 1994; Yang et al., 2014). According to the nutrient grading criteria of the second soil census in China (Shi et al., 2004; Yang et al., 2014), the soil fertility grades of the farmlands at our survey sites were all medium-low. After 2 years of field trips from 2019 to 2020, fifteen sites in six counties were finally identified as sampling areas for this study (Supplementary Table 1). Each site had both conventional and organic farmlands planted with maize, i.e., large areas of conventional farmland and small areas of organically farmed maize fields. A pair of sampling areas, one conventional (Con) maize field and one organic (Org) maize field, were randomly selected at each site, away from the road. Thus, a total of 15 sites with both farming systems were included in this study, for a total of 30 sampling areas.

All the conventional farmlands we investigated are under a continuous maize monoculture cropping system. In the past 4 years, the conventional tillage (rotary plowing and residue removal) in the study area was gradually replaced by conservation tillage (ridge tillage and residue retention) as recommended by the government (The Ministry of Agriculture and Rural Affairs of China, 2020). Chemical compound fertilizers and herbicides were used in conventional farmlands every year. The fertilizer applied in organic farming is generally based on animal manure and no herbicides were applied. Weeds were controlled manually in organic farmlands at the beginning of the growing season and then suppressed by the canopy of maize. Organic farmlands have been under organic farming for at least 5 years, and some of them are more than 30 years (Supplementary Table 1). According to our field survey, the maize varieties planted on conventional and organic farmlands not only varied from place to place but also from year to year. Basically, the type of corn on organic farms is waxy corn while dent corn is common on conventional farms in our sampling area.

### Sample Collection

All soil and root samples from the maize fields were collected in late August 2020 (reproductive stage of maize). Three sampling spots were randomly selected in each sampling field, with a distance of about 5–10 m between each sampling spot. After removing approximately 2 cm of topsoil, a soil volume of 20 cm × 20 cm × 30 cm was dug at a horizontal distance of around 15 cm from the maize stalk, and the fine roots of maize were picked out. The soil from the three sampling spots was mixed thoroughly, and then about 2 kg of fresh soil was randomly collected and stored in plastic sealed bags (soil sample I). About 10 g of fresh soil was randomly selected into plastic sealed bags and placed in an icebox (soil sample II). Fine maize roots from the three sampling spots were mixed and stored in plastic bottles containing formalin–acetic acid–alcohol (FAA) fixative. All soil and root samples were then transported back to the laboratory. Impurities such as plant and animal residues were removed from soil sample I and air-dried indoors to measure AM spore density and soil parameters. Soil sample II was stored in a refrigerator at −80°C for 3 days and then sent to Majorbio Co., Ltd., for DNA extraction and sequencing of AM fungi. FAA-fixed roots were used to measure the percentage and intensity of AM fungal colonization.

### Soil Parameters Measurement

The soil sample I was air-dried and sieved through a 40-mesh sieve (<0.425 mm). Soil pH was measured in a soil-water slurry (1:5, w/v) using a pH meter (Mettler-Toledo pH reader, Switzerland). The potassium dichromate method was used to determine the organic carbon content of the soil (Lu, 1999). The soil’s total nitrogen content was determined using the Kjeldahl method (Lu, 1999). The soil hydrolysable nitrogen was measured by alkaline hydrolysis diffusion (Lu, 1999). The total and available phosphorus contents were determined spectrophotometrically by a continuous flow analyzer (SAN + +, Skalar, Breda, Holland).

### Arbuscular Mycorrhizal Fungal Colonization and Spore Density Determination

The FAA-fixed roots were washed with 10% KOH solution at 90°C for 60 min, acidified with 1% HCl for 30 min, and then cut into approximately 1 cm root segments. The root segments were soaked in 0.05% (v) trypan blue solution and then placed on a hot plate at 90°C for 30 min (Koske and Gemma, 1989). The stained roots were mounted on microscope slides for assessment of AMF colonization following the method of Trouvelot et al. (1986). The percentage of colonization (frequency of the mycorrhiza in the root system) and colonization intensity (intensity of the mycorrhizal colonization in the root system) were then calculated (Trouvelot et al., 1986).

AMF spores were isolated from 20 g of randomly divided dry soil sample I by wet sieving and gradient centrifugation (Gerdemann and Nicolson, 1963). The spore density (total number of spores in 20 g of dry soil) was determined by the number of spores counted under a stereomicroscope (Daniels and Skipper, 1982). Only spores with complete shape structure were selected for counting.

### DNA Sequencing

#### DNA Extraction and Amplicon Sequencing

The total DNA of 0.5 g soil samples was extracted according to the manufacturer’s instruction using a E.Z.N.A.^®^ soil (Omega Bio-Tek, Norcross, GA, United States). The primers AML1F/AML2R were used to amplify the V4-V5 hypervariable regions of the fungal 18S rRNA gene in a thermocycler PCR system (Axygen Biosciences, Union City, CA, United States). The second amplification used the AMF specific primer set AMV4.5NF/AMDGR. The PCR reactions were conducted using the following program: 3 min of denaturation at 95°C, 27 cycles of 30 s at 95°C, 30 s for annealing at 55°C, and 45 s for elongation at 72°C, and a final extension at 72°C for 10 min. PCR reactions were performed in a triplicate 20 μL mixture containing 4 μL of 5 × FastPfu Buffer, 2 μL of 2.5 mM dNTPs, 0.8 μL of each primer (5 μM), 0.4 μL of FastPfu Polymerase and 10 ng of template DNA.

#### Bioinformatics

Raw FASTQ files were demultiplexed, quality-filtered by Trimmomatic, and merged by FLASH with the following criteria: (i) The reads were truncated at any site receiving an average quality score < 20 over a 50 bp sliding window. (ii) Primers were exactly matched allowing 2 nucleotide mismatching and reads containing ambiguous bases were removed. (iii) Sequences whose overlap was longer than 10 bp were merged according to their overlap sequence. Operational taxonomic units (OTUs) were clustered with a 97% similarity cutoff using UPARSE (version 7.1^1^) and chimeric sequences were identified and removed using UCHIME. The taxonomic identities of the representative sequences were checked against the Maarjam081 AMF database online (^2^ Accession to cite for these SRA data: PRJNA819886).

### Data Analysis

Four OTU diversity indices of AM fungal communities were calculated, including the Chao1 index, the Shannon diversity index, the Simpson evenness index, and the phylogenetic diversity index (Caporaso et al., 2010). The conventional and organic farms are matched sample pairs, therefore paired *t*-tests were performed using SPSS 25.0 software (IBM) to analyze whether soil parameters, mycorrhizal colonization, spore density, and diversity indices of AM fungi differed between conventional and organic farming systems. Normality and homogeneity of the distribution of residuals were verified and log or arcsine transformations were performed when necessary.

To detect differences in soil AM fungal community structure between conventional and organic maize farms, non-metric multidimensional scaling (NMDS) was performed using the R package “vegan” based on Bray–Curtis dissimilarities of the compositional data matrix. Differences in Bray-Curtis dissimilarity between the two farming systems were also Monte Carlo permutation tested for significance.

Using the “SpiecEasi” package in R software, OTU network maps of soil AM fungi from conventional and organic farming maize fields were constructed separately. Several parameters characterizing the networks were calculated. OTUs with relative abundance > 0.05% were selected for SparCC correlation analysis, and correlation networks were constructed by screening for significant (*P* < 0.01) and strong (| r| > 0.3) correlations (Kim et al., 2020). Network visualization was performed on the Gephi (v.0.9.2) platform^3^ and colored by family classification.

To investigate whether AM fungal colonization, spore density, and diversity indices were related to soil factors, correlation heat maps were drawn in two farming systems based on the Spearman correlation coefficients between five soil factors (pH, contents of organic matter, total and available nitrogen, total and available phosphorus) and the above indicators. Using the “gbmplus” package (R v.3.6.3) with 500 trees for boosting (De’ath, 2007), an Aggregated Boosted Tree (ABT) analysis was conducted to quantify the effect of five soil factors on the OTUs composition of AM fungal communities.

## Results

### Root Inoculation and Spore Density

**FIGURE 2:**
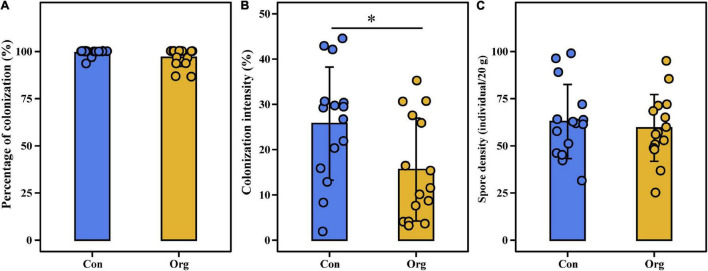
The percentage of colonization **(A)**, colonization intensity **(B)** of roots, and soil spore density **(C)** on conventional (Con) and organic (Org) maize farms. The * represents the difference between two farming systems is significant at *P* = 0.05 level.

The percentage of plant root colonization was less variable (86.67–100%), but colonization intensity (1.86–44.5%) and spore density (25–103 spores/20 g soil) were highly variable across all sampling areas including conventional and organic farms (Figure 2). Percentage of colonization (*t* = 1.733, *P* = 0.105) and spore density (*t* = −0.822, *P* = 0.425) were not different between the two farming systems (Figures 2A,C), but colonization intensity was different (*t* = 2.159, *P* = 0.049, Figure 2B).

### Arbuscular Mycorrhizal Fungal Community Diversity

**FIGURE 3:**
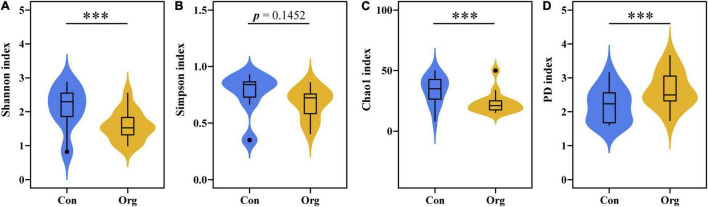
The OTU diversity indices of AM fungal communities on conventional (Con) and organic (Org) maize farms including Shannon diversity index **(A)**, Simpson evenness index **(B)**, Chao 1 index **(C)**, and phylogenetic diversity index **(D)**. The *** represents the difference between the two farming systems is significant at *P* = 0.001 level.

A total of 171 OTUs of AM fungi were detected in all samples, of which 107 and 125 OTUs were detected on conventional and organic farms, respectively. The diversity of soil AM fungal community was different between the two farming systems. The OTU Shannon diversity and Chao1 index of AM fungal communities were higher on conventional farms than on organic farms (Figures 3A,C), while the difference in Simpson evenness index was not significant (Figure 3B). The OTU phylogenetic diversity index (PD) was lower for AM fungal communities on conventional farms than on organic farms (Figure 3D).

### Arbuscular Mycorrhizal Fungal Community Composition and Network Analysis

**FIGURE 4:**
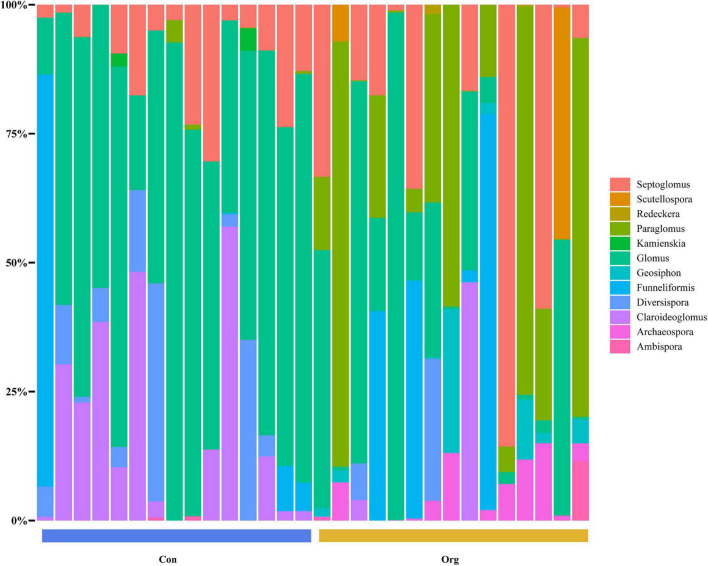
The genus composition of soil AM fungal communities on conventional (Con) and organic (Org) maize farms.

For maize fields in the conventional farming system, 64.79% of the AM fungi OTUs belonged to the family Glomeraceae (including 41.8% *Glomus*, 14.08% *Septoglomus*, 8.68% *Funneliformis*, and 0.23% *Kamienskia*), 13.88% to Paraglomeraceae (*Paraglomus*), 9.7% to Claroideoglomeraceae (*Claroideoglomus*), and 5.45% to Diversisporaceae (*Diversispora*). Other families, such as Archaeosporaceae (*Archaeospora*), Geosiphonaceae (*Geosiphon*), Gigasporaceae (*Scutellospora*), Ambisporaceae (*Ambispora*), and Diversisporaceae (*Redeckera*), represented less than 10% of the total OTUs (Figure 4).

For maize fields in the organic farming system, 54.73% of the AMF OTUs belonged to the family Glomeraceae (including 25.63% *Glomus*, 18.04% *Septoglomus*, 11.06% *Funneliformis*), 27.34% to Paraglomeraceae (*Paraglomus*). Other families (each was less than 5%), including Ambisporaceae (*Ambispora*), Archaeosporaceae (*Archaeospora*), Claroideoglomeraceae (*Claroideoglomus*), Diversisporaceae (*Diversispora*, *Redeckera*), Geosiphonaceae (*Geosiphon*), and Gigasporaceae (*Scutellospora*) were about 18% in total (Figure 4).

**FIGURE 5:**
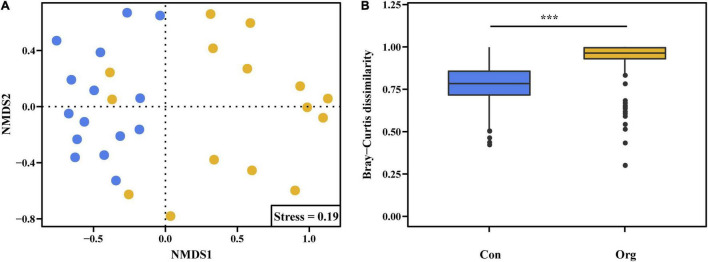
Non-metric multidimensional scaling ordinations **(A)** of AM fungal communities on conventional (blue) and organic (yellow) farms based upon their OTU composition derived from Bray-Curtis distances **(B)**. The *** represents the difference between the two farming systems is significant at *P* = 0.001 level.

Based on the results of the non-metric multidimensional scaling analysis, Bray-Curtis dissimilarity was different between the two farming systems, indicating that the OTU composition of soil AM fungal community differed between conventional and organic farms (Figure 5).

**FIGURE 6:**
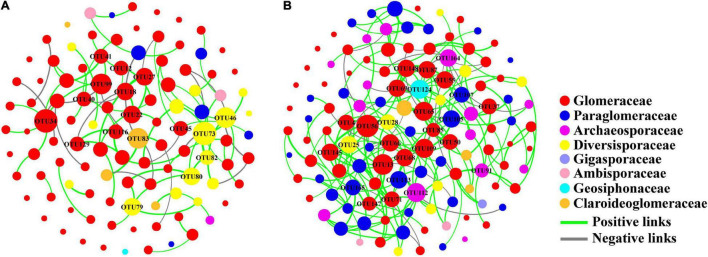
Network diagram of AM fungal community on conventional **(A)** and organic **(B)** maize farms. The size of each node is proportional to the number of connections (degree).

We found that the soil AM fungal network on both conventional (modularity of 0.814) and organic farms (modularity of 0.724) had a modular structure (>0.4) (Table 1). The nodal OTUs with high connectivity were predominantly from the Glomeraceae and Diversisporaceae families in the conventional farming system while from the Glomeraceae and Paraglomeraceae in the organic farming system (Figure 6). Furthermore, the key taxon of the Glomeraceae family included completely different OTUs components on conventional and organic farms, respectively.

**TABLE 1:** The network attributes of AM fungal community composition on conventional (Con) and organic (Org) maize fields.

Attributes	Con	Org
Number of nodes	107	125
Number of links	88	206
Average degree	1.645	3.296
Average weighted degree	0.963	1.918
Graph density	0.016	0.027
Modularity	0.814	0.724
Average clustering coefficient	0.227	0.238
Average path length	7.106	4.937

The soil AM fungal network of organic farms was more complex because it had more nodes and links, higher average connectivity, graph density, and average clustering coefficients (Table 1 and Figure 6). Some negative connections between nodes appeared in the network of conventional farms (21.59% of total links) and organic farms (12.14% of total links).

### Soil Factors

Except for soil pH, the soil organic matter, total nitrogen, total phosphorus, available nitrogen and phosphorus contents were higher on organic farms (Supplementary Table 2). In particular, the difference in soil available phosphorus content between the two farming systems was about eight-fold.

**FIGURE 7:**
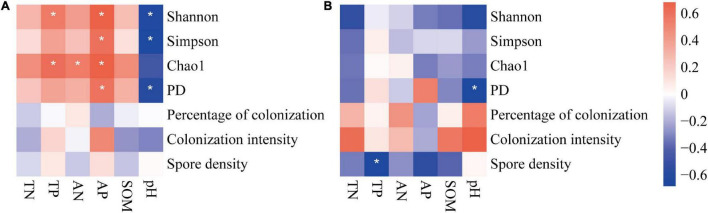
The correlation between soil factors and indices of AM fungal communities on conventional **(A)** and organic **(B)** maize farms. The *, **, and *** represents the correlation is significant at *P* = 0.05, 0.01, and 0.001 level, respectively.

The correlations between soil factors and soil AM fungi (colonization, spore density, and diversity) were different in the two farming systems (Figure 7). In general, soil factors were correlated with AM fungal diversity on conventional farms, while they were hardly significant on organic farms. On conventional farms, all four diversity indices of soil AM fungi increased with increasing soil nutrients (especially phosphorus) content and decreasing soil pH (Figure 7A). Only the phylogenetic diversity index was negatively correlated with soil pH on organic farms (Figure 7B). In all farming systems, neither the percentage nor the intensity of root colonization was correlated with soil factors. On organic farms, the spore density was negatively correlated with total and available soil phosphorus content (Figure 7B).

**FIGURE 8:**
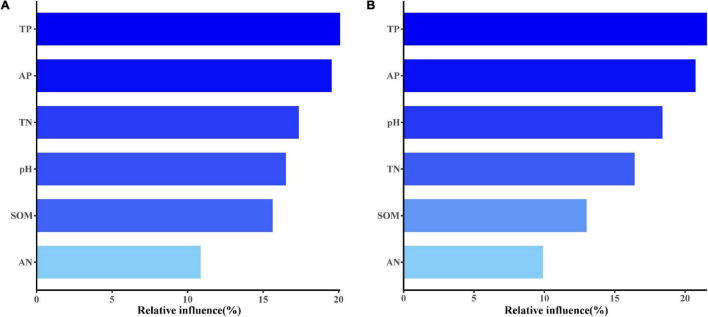
The aggregated boosted tree (ABT) analysis showed the relative influence of each soil factor on AM fungal community composition on conventional **(A)** and organic **(B)** maize farms.

On both conventional and organic farms, total soil phosphorus and available phosphorus content were the most important factors affecting soil AM fungal community composition (Figure 8). In addition to phosphorus, total soil N content on conventional farms and soil pH on organic farms also influenced the community composition of AM fungi in maize fields (Figure 8).

## Discussion

### Higher Colonization Activity of Arbuscular Mycorrhizal Fungi on Conventional Farms

There is a generally accepted opinion that conventional farming systems inhibit AM fungal colonization, while organic farming systems enhance AM fungal colonization to plant roots (Cely et al., 2016; Jiang Y. J. et al., 2020). However, in the present study, we have found the opposite result in that colonization intensity was higher on conventional farms (Figure 2B). Furthermore, soil nutrients have been verified in many previous studies as the main factor influencing AM fungal colonization and reproduction (Johnson, 2010; Begum et al., 2019). But our study showed that although the soil nutrient content was higher on organic farms than on conventional farms (Supplementary Table 2), the percentage and intensity of colonization and spore density were independent of almost all the soil factors we measured (Figure 7). The different results found in this study may be due to the adaptation of the AM fungal taxa to the nutrient levels in the low-quality of soil in the area.

According to the nutrient grading criteria of the second soil census in China (Zhu, 1994; Shi et al., 2004), all soil fertility classes in the area were medium-low. Regardless of whether the input nutrients are chemical or organic fertilizers, soil nutrient content can only increase over a certain period and within a limited soil space (Cavagnaro et al., 2015; Yang et al., 2017). In this study, almost all roots sampled from conventional and organic farms were colonized by AM fungi (Figure 2A), suggesting that crop plants may rely on the cooperation of AM fungi for additional resources. This is consistent with the idea that plant-AM fungi symbiosis is easily established under low nutrient conditions (Graham and Eissenstat, 1994; Lekberg et al., 2021). In addition, the nutrient content of conventional field soils is more likely to fall back to the original low-quality level due to crop uptake of nutrients released rapidly from chemical fertilizers or nutrient loss by leaching (Bender and van der Heijden, 2015). The nutrient content of conventional field soils during a later growth season (late August) was lower than that in organic field soils, which might contribute to the increased colonization intensity on conventional farms in this study.

Therefore, background soil fertility levels, as well as other environmental factors (e.g., salinity, drought, etc.), should be considered when we assess the effects of conventional and organic farming systems on soil AM fungi (Franke-Snyder et al., 2001; Boomsma and Vyn, 2008). Because their strong effects on AM fungi may interfere with our correct understanding of the beneficial and detrimental effects of different farming systems on the ecological functions of AM fungi and/or other soil organisms.

### Lower Phylogenetic but Higher Taxonomic Diversity of Arbuscular Mycorrhizal Fungal Operational Taxonomic Unit on Conventional Farms

Conventional and organic farming systems not only differently affect the establishment of the plant-AM fungi symbiosis, but also result in different diversity of soil fungal communities (Sale et al., 2015; Roy et al., 2017). It is generally accepted that the diversity of soil AM fungal communities at the family or genus level is often decreased when farmland is subjected to high levels of chemical fertilizers, herbicides, and mechanized tillage under conventional farming management (Zeng et al., 2021). Furthermore, the most common taxon of AM fungal community in such farming systems tends to be *Glomus*, a more disturbance-tolerant and widely distributed genus (Daniell et al., 2001; Hart and Reader, 2002). Our study is consistent with this (Figure 4). However, although fewer families and genera were found on conventional farms, their OTU diversity was higher than on organic farms (Figures 3A,C).

Soil fertility was low to medium on both conventional and organic farms (Zhu, 1994; Shi et al., 2004), but soil nutrient levels were higher on organic farms than on conventional farms, especially for available phosphorus content (Supplementary Table 2). Livestock manure is the main component of organic fertilizer in the region, which contains high phosphorus content (Liu et al., 2019). The phosphorus content is an important factor in establishing symbiosis and AM fungal diversity (Xiao et al., 2019). Usually, the increase in AM fungal diversity with increasing soil phosphorus happens in low fertile soils (Johnson et al., 2003), till a threshold level beyond which phosphorus negatively affects AM fungi (Kiers et al., 2011; He et al., 2016). Relatively adequate P availability in nutrient-poor soils may reduce plant dependence on AM fungi, which in turn may reduce carbohydrate supply to fungi from the root system (Jakobsen et al., 1992; Kiers et al., 2011). Thus, higher soil nutrient levels, especially P content, on organic farms may be one of the reasons for their lower OTU diversity.

On the other hand, since different nucleotypes carrying different genetic information may rapidly diverge (Angelard et al., 2014), the nucleotypes adapted to conventional farm soil conditions (stable soil disturbance, drought, and salinity) then subsequently accumulate and occupy the ecological niches left by those that were filtered out (long-term studies are needed to ascertain this). This allows an AM fungus to undergo rapid genotypic change by altering the relative frequencies of different nucleotypes in response to environmental change and results in high functional diversity (Munkvold et al., 2004; Kruger et al., 2012). Therefore, although only relatively few families were detected on conventional farms, the OTU diversity of AM fungi was high (Figures 3, 4). This has an important implication for mycorrhizal inoculant production and application, as the AM fungal OTUs currently present in low-quality or stressed agricultural fields may be potential and valuable resources. Compared to common commercial mycorrhizal agents, native strains screened from local environments are more effective for plant-fungi symbiosis (Chenchouni et al., 2020; Wu et al., 2021). In addition, fungal inoculants using native strains may be more beneficial in maintaining or restoring stable soil micro-food webs.

### Simple and Different Network Structures of Arbuscular Mycorrhizal Fungi on Conventional Farms

As some microbial groups are more sensitive to environmental disturbances than others (Bhadalung et al., 2005; Qin et al., 2015), not only the diversity of soil AM fungi, but also the community structure may be altered by agricultural management on conventional farmland (Bhadalung et al., 2005; Chen et al., 2014). *Glomus* is often considered to be the most common fungal genus in agricultural soils (Berthrong et al., 2013). Our data showed that *Glomus* comprised 41.8% of the AM fungal community on conventional farms, higher than the 25.63% on organic farms (Figure 4). Conventional commercial farmland is subjected to more mechanized tillage than organic farmland, and some of the reproductive characteristics of *Glomus*, such as the ability to reproduce not only through spores but also through mycelial fragments (Biermann and Linderman, 1983), mitigate to some extent the negative effects of frequent tillage on the survival of AM fungi. These reproductive properties and the widely known stress tolerance give *Glomus* an advantage in AM fungal communities that suffer from various agricultural disturbances (Hijri et al., 2006; Brito et al., 2012).

In contrast to the decreasing proportion of *Glomus* in soil AM fungal community, *Paraglomus* increased on organic farms and even became the most common genus in some sample sites (Figure 4). Several studies have found that the genus occurs mainly on organic rather than conventional agricultural soils (Gosling et al., 2014). Less disturbing agricultural management and higher soil phosphorus levels may have contributed in part to the increase of *Paraglomus* on organic farmland (Jiang S. et al., 2020). The dominant family in conventional and organic farming systems was the same, i.e., Glomeraceae.

Network analysis further suggested that the dominant taxon Glomeraceae was also a keystone taxon in the network structure of soil fungi, regardless of the farming system. Keystone taxon has a unique position in microbial communities and their absence or alteration has a significant impact on community structure and function (Banerjee et al., 2018). Although Glomeraceae is the key taxon of the fungal network in both farming systems, this family included completely different OTUs components on conventional and organic farms, respectively (Figure 6). This meant that the genetic composition of the dominant Glomeraceae was completely different, which may lead to different functions of the soil AM fungal community in these two farming systems. Furthermore, the conventional farm management has led to a simpler network structure of soil AM fungal communities and weaker interactions between OTU nodes. The higher percentage of negative links on conventional farms may imply more competitive interactions or niche differentiation among OTUs (Deng et al., 2016). Despite the high OTU diversity of soil AM fungal communities on conventional farms, the simpler network structure may reduce their functions and resistance to environmental changes compared to organic farms.

## Conclusion

Comparing the differences in soil AM fungal community composition between conventional and organic farms is important for both agriculture production and restoration of degraded arable soil. We found a higher colonization intensity of maize roots by AM fungi and higher OTU taxonomic diversity on conventional farms compared to organic farms, which was contrary to what had been found. This suggests that the background soil fertility level should be considered when assessing the effects of conventional and organic farming systems on soil AM fungi. Though conventional farming systems resulted in different compositions and simpler structures of soil AM fungal community, there are potential diverse OTU resources currently present on conventional farms, which might be valuable for efficient mycorrhizal inoculant production. The AM fungal strains screened from conventional farms may be potential strains for creating more efficient biofertilizers for industrial farmland applications, while strains from organic farms may be a potential resource for degraded arable land fauna recovery or sustainable agriculture.

## Data Availability Statement

The datasets presented in this study can be found in online repositories. The names of the repository/repositories and accession number(s) can be found in the article/Supplementary Material.

## Author Contributions

PW and YZ contributed to the study’s conception and design. JC and YW performed the material preparation and data collection. JC, JL, and YY performed the data analyses. JC and JL wrote the first draft of the manuscript. All authors commented on previous versions of the manuscript, read, and approved the final manuscript.

## Conflict of Interest

The authors declare that the research was conducted in the absence of any commercial or financial relationships that could be construed as a potential conflict of interest.

## Publisher’s Note

All claims expressed in this article are solely those of the authors and do not necessarily represent those of their affiliated organizations, or those of the publisher, the editors and the reviewers. Any product that may be evaluated in this article, or claim that may be made by its manufacturer, is not guaranteed or endorsed by the publisher.

## Footnotes

1
http://drive5.com/uparse/

2
http://maarjam.botany.ut.ee/

3
https://gephi.org/
