# Supplementary material for: Effects of Conventional and Organic Agriculture on Soil Arbuscular Mycorrhizal Fungal Community in Low-Quality Farmland
Source: Front Microbiol. 2022 Jun 9;13:914627. doi: 10.3389/fmicb.2022.914627 (PMC9218867; doi:10.3389/fmicb.2022.914627)
Supplement: Supplementary file 1 [file Table_1.DOCX]

Supplementary Material

Supplementary Table 1. The 15 investigated sites of western Jilin Province, China. Each site had both conventional and organic farmland planted with a continuous maize monoculture cropping system.

| Number | County | Site | Longitude and Latitude | Years of organic farming |
| --- | --- | --- | --- | --- |
| 1 | Baicheng | Honggangzi | 45°36′53″N，123°55′52″E | 5 |
| 2 | Baicheng | Lisheng | 45°22′18″N，123°17′14″E | 8 |
| 3 | Taonan | Bajianfang | 45°34′38″N，123°01′22″E | 5 |
| 4 | Taonan | Haolibao | 45°15′09″N，122°49′26″E | 5 |
| 5 | Tongyu | Shuanggang | 45°06′00″N，122°55′48″E | >10 |
| 6 | Tongyu | Lengjiadian | 44°43′37″N，122°46′05″E | 5 |
| 7 | Tongyu | Wulanhua | 44°43′00″N，122°38′23″E | 5 |
| 8 | Tongyu | Xinglongshan | 44°49′39″N，122°26′148″E | >30 |
| 9 | Qianan | Lanzi | 44°50′02″N，123°31′39″E | 5 |
| 10 | Qianan | Caizi | 44°56′04″N，123°50′52″E | >20 |
| 11 | Qianan | Erlongshan | 45°02′34″"N，124°28′59″E | 8 |
| 12 | Qianan | Gudian | 44°53′57″N，124°35′11″E | >10 |
| 13 | Qianguo | Wulan | 44°33′57″N，124°22′26″E | >10 |
| 14 | Qianguo | Xiboyin | 44°21′07″N，124°04′20″E | >30 |
| 15 | Changling | Xinyicun | 44°02′58″N，123°52′17″E | >10 |

Supplementary Table 2. The soil properties (Mean ± SE) on conventional (Con) and organic (Org) maize fields and the results of paired t-test

| Soil properties | Con | Org | *t* | *P* |
| --- | --- | --- | --- | --- |
| pH | 8.30±0.10 | 8.29±0.10 | 0.035 | 0.973 |
| Organic matter (%) | 1.06±0.10 | 1.59±0.21 | -3.436 | 0.004 |
| Total nitrogen (g/kg) | 0.79±0.08 | 1.04±0.12 | -4.155 | 0.001 |
| Total phosphorus (g/kg) | 0.40±0.03 | 0.58±0.05 | -2.794 | 0.014 |
| Available nitrogen (mg/kg) | 61.54±6.06 | 89.88±11.38 | -3.139 | 0.007 |
| Available phosphorus(mg/kg) | 10.28±2.64 | 87.64±15.05 | -4.779 | < 0.001 |
